# Prediction and Characterisation of the System Effects of Aristolochic Acid: A Novel Joint Network Analysis towards Therapeutic and Toxicological Mechanisms

**DOI:** 10.1038/srep17646

**Published:** 2015-12-01

**Authors:** Wenna Nie, Yana Lv, Leyu Yan, Xi Chen, Haitao Lv

**Affiliations:** 1 Chongqing University Innovative Drug Research Centre, School of Chemistry and Chemical Engineering, Chongqing 401331, P.R. China; 2 Yunnan Branch, Institute of Medicinal Plant Development, Chinese Academy of Medical Sciences, Peking Union Medical College, Jinghong 666100, P.R China; 3 Key Laboratory of Dai and Southern Medicine of Xishuangbanna Dai Autonomous Prefecture, Jinghong 666100, P.R. China; 4 Institute of Medicinal Plant Development, Chinese Academy of Medical Sciences, Peking Union Medical College, Beijing 100193, P.R. China; 5 Tissue Repair and Regeneration Program, Institute of Health and Biomedical Innovation, Queensland University of Technology, Brisbane, QLD 4059, Australia

## Abstract

Aristolochic acid (AA) is the major active component of medicinal plants from the Aristolochiaceae family of flowering plants widely utilized for medicinal purposes. However, the molecular mechanisms of AA systems effects remain poorly understood. Here, we employed a joint network analysis that combines network pharmacology, a protein–protein interaction (PPI) database, biological processes analysis and functional annotation analysis to explore system effects. Firstly, we selected 15 protein targets (14 genes) in the PubChem database as the potential target genes and used PPI knowledge to incorporate these genes into an AA-specific gene network that contains 129 genes. Secondly, we performed biological processes analysis for these AA-related targets using ClueGO, some of new targeted genes were randomly selected and experimentally verified by employing the Quantitative Real-Time PCR assay for targeting the systems effects of AA in HK-2 cells with observed dependency of concentration. Thirdly, the pathway-based functional enrichment analysis was manipulated using WebGestalt to identify the mostly significant pathways associated with AA. At last, we built an AA target pathway network of significant pathways to predict the system effects. Taken together, this joint network analysis revealed that the systematic regulatory effects of AA on multidimensional pathways involving both therapeutic action and toxicity.

Aristolochic acid (AA) is an active compound that is derived from medicinal plants of the Aristolochiaceae family and has been broadly utilised for medicinal purposes for thousands of years. AA is a mixture of structurally related nitrophenanthrene carboxylic acids, with aristolochic acid I (AAI) and aristolochic acid II (AAII) is regarded as the major active components of AA^1^. The AA-containing drugs derived from these medicinal plants are often used in obstetrics and for treating snake bites, cancer, microorganisms, type B hepatitis, inflammation, arthritis and rheumatism^1^,^2^,^3^,^4^,^5^. However, AA has been also identified as a strong cytotoxic nephrotoxin and carcinogen in terms of its toxicity to complex systems, thus limiting its clinical application and resulting in severe host side effects termed as aristolochic acid nephropathy (AAN), which is a chronic, fibrosing, interstitial nephritis disease^1^,^6^. AAN was first reported in Belgium in 1993, and soon afterwards, similar cases were found in Asia and in other European countries^2^,^7^,^8^,^9^. A high risk of urothelial cancer was also diagnosed with AAN patients^2^,^10^. Some reports have indicated that AA exposure is the primary cause of AAN and Balkan endemic nephropathy, which results in the formation of specific AA-DNA adducts and in the mutation and overexpression of TP53, affecting the development of AAN-associated urothelial cancer^11^,^12^. AA is also known to be a potent mutagen and carcinogen, being among the most potent 2% of carcinogens^13^. Thus, herbal remedies containing AA have been classified as human carcinogens by the International Agency for Research on Cancer (IARC)^14^. In wide-range clinical trials, AA primarily induced multiple carcinomas, which were found in the stomach, liver, kidney, bladder, lung, skin and other organs. Due to the nephrotoxic and carcinogenic effects of AA, many associated pharmaceutical products have been banned in many Western countries. Moreover, AA further contributes to multiple forms of toxicity such as renal tubular epithelial cell degeneration, necrosis, apoptosis, dysregulated prostaglandin metabolism, genotoxic, and reproductive toxicity because of its bioactivities and reactions with cellular proteins and DNA^15^,^16^,^17^,^18^. However, the toxicological targets and associated molecular mechanisms of AA remain unclear. Currently, a comprehensive method that can identify the targets of AA toxicity and effects, more importantly, that can explore relevant toxicological and therapeutic mechanisms of AA is necessary.

In recent years, network pharmacology, a systems biology-based methodology, has been utilised extensively for the study of traditional Chinese medicine (TCM)^19^,^20^. A newly emerged “TCM network pharmacology approach” derived from network pharmacology is providing a new opportunity for translating TCM from an experience-based medicine into an evidence-based medicine system, quickening TCM-based drug discovery, and enhancing current strategies for drug discovery^21^,^22^. The TCM network pharmacology approach is being broadly explored and exploited for the study of single herbs, medicine pairs, and TCM formulas^23^ by coordinating with the conventionally experimental methods. For example, Li *et al.*^24^, efficiently applied this approach to identify molecular targets and to determine the pharmacological mechanisms of a typical traditional Chinese medicine formula, Liu Wei Di Huang (LWDH). Moreover, network pharmacology strategy emphasises maximising drug efficacy and minimising drug side effects by targeting multiple channels of the signalling pathway^25^,^26^,^27^. This approach focuses on the toxic reaction of a specific component in a complex system and offers assistance for drug safety assessment^28^. Fan *et al.*^29^ reconstructed the network model to describe toxicological properties using the network pharmacology method, which provides a wealth of information for screening toxic substances and for determining the potential toxicity of known compounds in a complex biological system. Therefore, to the best of our knowledge, network pharmacology is supposed to be a promising method for better understanding the systems effects (systems toxicity) of AA against the biological systems.

In this study, we attempted to clarify AA toxicity and associated biochemical actions by employing network pharmacology method as a joint network analysis was employed for the first instance that effectively combined network pharmacology, a protein–protein interaction (PPI) database and functional annotation analysis to clarify.

## Materials and Methods

**Figure 1:**
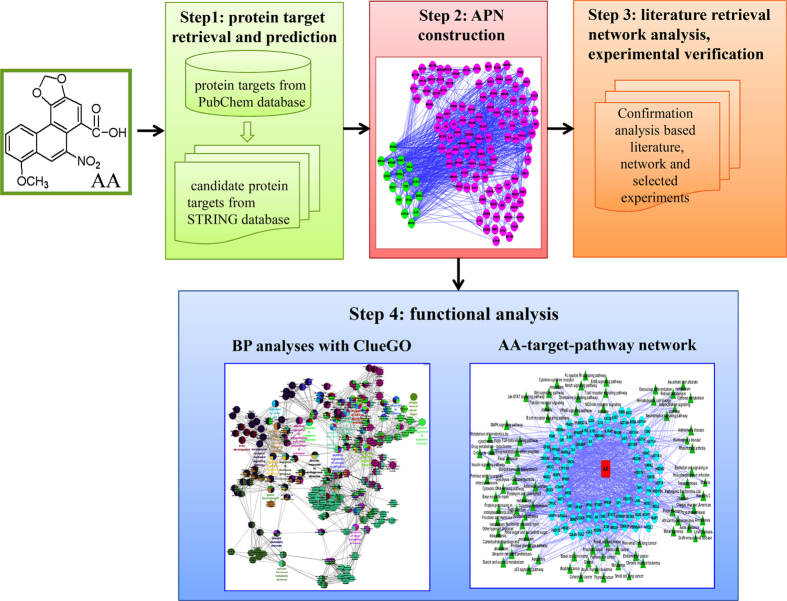
Schematic illustration of the standard workflow utilized in this study. This workflow is composed of the following four steps. 1) Retrieve protein targets from the PubChem database and their interaction proteins from the STRING database. 2) Visualise the AA-specific gene network (APN) using Cytoscape v 2.8.2. 3) Validate the genes associated with AA through literature retrieval, network analyses and experimental verification. 4) Engage in functional enrichment analysis of biological processes (BPs) and pathway analysis.

The standard flowchart this study is illustrated in Fig. 1, as we employed a joint network analysis that combines network pharmacology, a protein–protein interaction (PPI) database, biological processes analysis and functional annotation analysis to explore system effects. Firstly, we selected 15 protein targets (14 genes) in the PubChem database as the potential target genes and used PPI knowledge to incorporate these genes into an AA-specific gene network that contains 129 genes. Secondly, we performed biological processes analysis for these AA-related targets using ClueGO, 13 of new identified genes were randomly selected and experimentally verified by employing the Quantitative Real-Time PCR assay for the first time that noticeably characterized the systems effects of AA in HK-2 cells with observed dependence of concentration. Thirdly, the pathway-based functional enrichment analyses was manipulated by adopting WebGestalt to identify the mostly significant pathways associated with AA. At last, we built an AA target pathway network of significant pathways to predict and characterise the system effects.

### Retrieval of candidate protein targets

First, potential protein targets and their interaction proteins were retrieved from two independent sources, *i.e.*, the PubChem and STRING databases, respectively. PubChem (https://pubchem.ncbi.nlm.nih.gov/)^30^ is a public repository of small molecules and their biological properties, with more than 25 million unique chemical structures and 90 million bioactivity outcomes associated with several thousand macromolecular targets. The search for candidate protein target was manipulated using “aristolochic acid” as the query keyword, and a list of protein targets was generated. This initial protein target list was prioritised based on different species and different active compounds against the protein targets. We selected protein targets from *Homo sapiens* with active compounds ≥ 1 and acquired a shortlist of potential protein targets.

Second, the shortlist of potential protein targets, Table 1, was used as the seed to search for direct and indirect interacting proteins by subjecting this list to STRING 9.1 database searches^31^. STRING is a database that is composed of the known and predicted relationships of protein interactions and that provides a wealth of biochemical information from widely different sources regarding their neighbourhood, gene fusion, co-occurrence, co-expression, experiments, database and literature mining.

Finally, for each seed target, we aimed at determining its direct interacting protein as a candidate protein target. Let S be defined as a set that contains all seed targets and their direct interacting proteins. Here, we employed STRING to predict interactions between and among seed targets and their direct interacting proteins in S.

### Network construction of AA targets

Firstly, we combined all potential protein targets and candidate protein targets to construct an AA-specific gene network. Secondly, this gene network was further visualised using Cytoscape v 2.8.2 software^32^. Notably, we adopted the STRING database to predict interactions between potential protein targets and interactions among candidate protein targets.

### Functional annotation of AA-related targets

#### Biological processes analysis

To further understand the biological relevance of the characterized targets related to AA, we performed biological processes analysis using ClueGO^33^. The Cytoscape plug-in ClueGO is a professional software that was widely employed to facilitating the visualisation of functionally related genes, which are characterized and displayed as a clustered network and as a statistical chart as the ClueGO setting term: “Biological processes” was selected in this analysis. The statistical test used for the enrichment was based on a two-sided hypergeometric option with a Bonferroni correction, a P-value less than 0.001 and a kappa score of 0.35. In addition, the medium network type was selected as well. To establish the annotation network, functional groups were visualised in the network via ClueGO assay, which primarily adopted the organic layout algorithm.

#### Network mode construction of AA target pathways

We performed the pathway-based functional enrichment analyses using WebGestalt (Web-based Gene Set Analysis Toolkit, http://genereg.ornl.gov/webgestalt)^34^. A P-value less than 0.05 was considered the cut-off criterion. WebGestalt is a system that facilitates the analysis of sets of genes that can be visualised and organised by a user-selected method (*e.g.*, Gene Ontology, KEGG Pathway), and different annotations can be selected and retrieved for each set. Here, we constructed an AA target pathway network by connecting AA, predicted targets and their pathways. At last, the AA target pathway network was visualised using Cytoscape v 2.8.2.

#### HK-2 cells exposure to AAI treatment

HK-2 cell was originally purchased from (ATCC, USA) and maintained in DMEM/F-12 at 37 °C with 5% CO2, cells were seeded in 10 mm dishes at a density of approximate 1 × 10^7^ cells per dish and then incubated for 12 h either without or with AAI at various concentrations as 10 mM, 50 mM and 100 mM, respectively.

#### Quantitative Real-Time PCR assay of the selected gene expressions in HK-2 cells treated by AA

Real-time PCR assay was carried out as the standard protocol reported by Mouritzen *et al.* (Mouritzen *et al.*, 2005) with minor modifications. Total RNAs were isolated from Human’s HK-2 cells using TRIZOL Reagent (Invitrogen Cat.No.15596-026) by referring to the manufacturer’s instruction. The QuantiTect RT Reverse Transcription Kit (Takera: Cat. No.RR047A) was utilized for cDNA amplification by according to the manufacturer’s protocol. The Real-time PCR Kit (Takera SYBR Premix Ex Taq II Cat. No. RR820A) was used to facilitate the sequence amplification by following the manufacturer’s guideline. To analyze the relative abundance of transcripts using quantitative RT-PCR, HK-2 cells were seeded in 10 mm dishes at a density of 1 × 10^7^ cells per dish to isolated RNA.1ug total RNA was reverse transcribed for 15 min at 37 °C to attain 20ul cDNA. Quantitative RT-PCR was performed in optical 96-well plates using Roche Light Cycle 96 three step Real-Time PCR systems (n = 3, triplicate repetition). Reactions were performed in a final volume of 25 μL containing 12.5 μL of SYBR premix Ex Taq II, 2 mMof each primer, and 10 ng of cDNA. PCR conditions were set as 95 °C for 30 s, followed by 45 cycles of 95 °C for 5 s and 60 °C for 30 s, 72 °C for 30 s. Fluorescence threshold data (Ct) was analyzed using Light Cycle 96 system software, then exported to Microsoft Excel for further plotting and visualization. Relative expressional levels in each cDNA sample were normalized to a NAPDH gene. In this study, the primer of each gene were designed using Primer 5 software. The sequence details were listed in Table 2. NAPDH gene was adopted as an internal control and to assess the efficiency of cDNA synthesis. Quantitative data was representative of the triplicate experiments.

## Results and Discussion

### Construction of an AA-specific gene network

**Figure 2:**
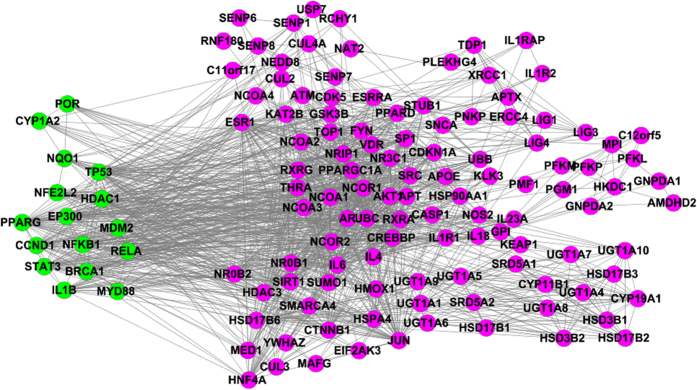
AA-specific protein network. Green denotes genes that can directly associate with AA. Pink denotes genes that can indirectly associate with AA.

We retrieved 15 potential protein targets (14 target genes) from the PubChem database (see Table 1) and 115 candidate protein targets (target genes) from the STRING database. The 129 genes were globally evaluated using the STRING database to characterise the interactions among them. As a result, an AA-specific protein network was constructed using the STRING database and then visualised using Cytoscape. This network involved 129 gene and 1250 interactions (Fig. 2). All genes in this network can be either directly or indirectly associated with AA according to literature retrieval and relevant network analyses.

Among the 129 genes involving potential protein targets of AA, 12.4% have a direct relationship with AA that was reported by previous studies. For example, TP53, a tumour suppressor gene, plays a pathogenic role in acute AAN, and p53 protein overexpression is closely linked to urothelial carcinomas and urothelial atypia in patients with AAN^35^,^36^. Moreover, STAT3, which is a signal transducer and transcription activator, also plays important roles in many cellular processes such as cell growth and apoptosis^37^,^38^. A study reported that AA induces TEC death *via* apoptosis by STAT3 dephosphorylation and by posttranslational p53 activation^39^. MDM2, a target gene of the transcription factor p53, is involved in cell cycle regulation^40^. Chen *et al.*^41^ and Volker *et al.*^42^ demonstrated MDM2 is significantly downregulated by AA treatment. NQO1 (NAD(P) quinone oxidoreductase), which is an AA-activating enzyme, might play a key role in cancer susceptibility due to AA exposure. In addition, Katerina *et al.*^43^ and Bárta *et al.*^44^ suggested that AAI can augment its own metabolic activation by inducing NQO1, thus heightening its own genotoxic potential. Also, BRCA1 is a known tumour suppressor. BRCA1 and/or p53 modulate AAI-induced genes involved in DNA damage and cell cycle regulation in renal tubular epithelial cells *in vitro*, and several important targets for prostate cancer are modulated by BRCA1 and p53^45^,^46^.

Moreover, 40% of the identified target genes are involved in many important mechanisms of action associated with AA. For examples, ATM, which is a regulator of p53 and BRCA1, also functionalizes as a pro-apoptotic gene involved in urothelial cancer cell apoptosis^47^,^48^. C12orf5 (TIGAR) is a p53-inducible gene that basically regulates glycolysis and apoptosis^49^. TIGAR increases NADPH production so as to limit the generation of reactive oxygen species (ROS) by beneficially modulating the pentose phosphate pathway^50^. TIGAR protein expression also protects cells from ROS-induced DNA damage and provides protection against DNA damage-induced apoptosis^51^,^52^. APOE, a lipid metabolism-related gene, is a protective factor for renal diseases^53^. These genes may be involved in many important therapeutic actions that indirectly correlate with AA, involving anti-inflammation, anti-cancer, anti-microorganism, apoptotic and fibrotic effects.

Other identified genes might indirectly contribute to the better understanding of system effects of AA. For example, Cdk5 is a mediator of neuronal death and the DNA damage response^54^. LIG1 mutations lead to immunodeficiency and to increased sensitivity to DNA-damaging agents^55^. MED1 regulates p53-dependent apoptosis and is essential for adipogenesis^56^. AA induces DNA damage, TP53 mutation or overexpression, and apoptosis and inhibits adipose accumulation^57^. However, there was no present evidence to manifest if those identified genes are actually correlated with systems effects of AA involving therapeutic actions and tissue toxicities.

**Figure 3:**
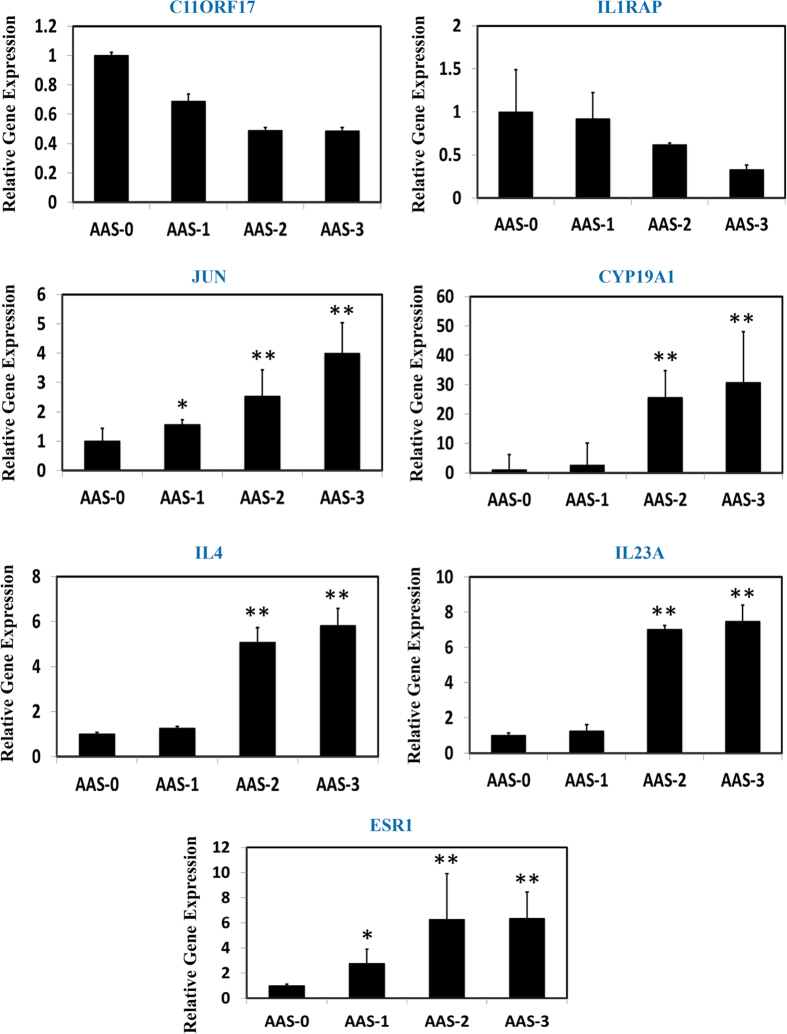
AAI treatment remarkable regulated the gene expressions with a remarkable concentration dependent in HK-2 cells. AAI-0: HK-2 cells without AAI treatment; AAI-1: HK-2 cells were treated with AAI (10 mM); AAI-2: HK-2 cells were treated with AAI (50 mM); AAI-3: HK-2 cells were treated with AAI (100 mM). Compared to AAS-0 (control group), it was considered as statistical difference while P value is less than 0.05 (*), and it was considered as significantly statistical difference when P value is less than 0.01 (**).

**Figure 4:**
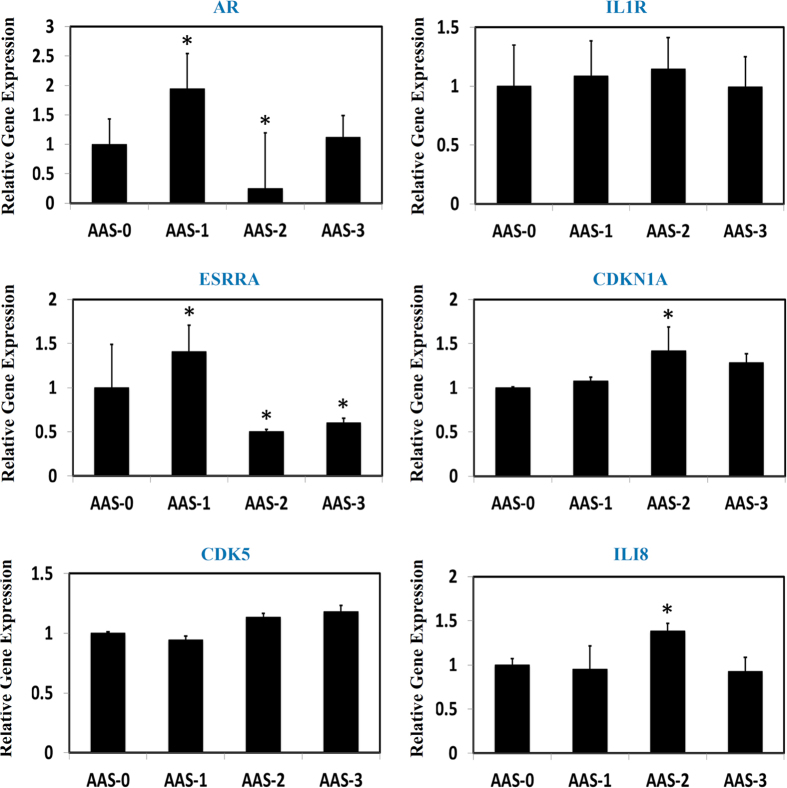
AAI treatment regulated the gene expressions in a variety of manners in HK-2 cells. AAI-0: HK-2 cells without AAI treatment; AAI-1: HK-2 cells were treated with AAI (10 mM); AAI-2: HK-2 cells were treated with AAI (50 mM); AAI-3: HK-2 cells were treated with AAI (100 mM). Compared to AAS-0 (control group), it was considered as statistical difference while P value is less than 0.05 (*), and it was considered as significantly statistical difference when P value is less than 0.01 (**).

To validate the predictive efficiency of the adopted joint network approach to the non-evident genes, we further treated the HK-2 cells with AAI in a variety of concentrations as 10 mM, 50 mM and 100 mM, respectively, then Quantitative Real-Time PCR assay was employed for the determination of relative expressional levels of 13 identified genes without direct evidence to link to the systems effects of AA against HK-2 cells, they are C11ORF17, IL1RAP, JUN, CYP19A1, IL4, IL23, ESR1, AR, IL1R1, ESRRA, CDKN1A, CDK5 and ILI8 (see Table 2). The results demonstrated that the normal expressional levels of the selected genes were significantly perturbed by AAI treatment in HK-2 cells, but not the IL1R1, CDK5 genes (see Figs 3 and 4). C11ORF17 and IL1RAP were down-regulated considerably when HK-2 cells exposed to AAI, however JUN, CYP19A1, IL4, HIL23A and ESR1 were observably upregulated by AAI. Interestingly those gene expressions were perturbed by AAI treatment rendered a remarkable dependent of concentration (Fig. 3). Contrast to the linear modulation of those identified genes by AAI intervention, the others including AR, ESRRA, CDKN1A and ILI8 were perturbed markedly by AAI treatment as well, while the expressional changes were not characterized as the noticeable dependent of concentration (Fig. 4). Take altogether, most of those selected genes were modulated significantly by AAI treatment that is consistent with our predictive results by the joint network approach as an experimental evidence confirmed and validated that this adopted network biology approach was feasible and confident, it holds the capacity to efficiently discovery and identify the candidate genes implicated in the defined biological events such as drug toxicity, drug effects or even disease development, which might provide novel insights into those biological events by integrating with relevantly experimental verification.

### Biological annotation of AA-related targets

#### Analysis of biological processes

**Figure 5:**
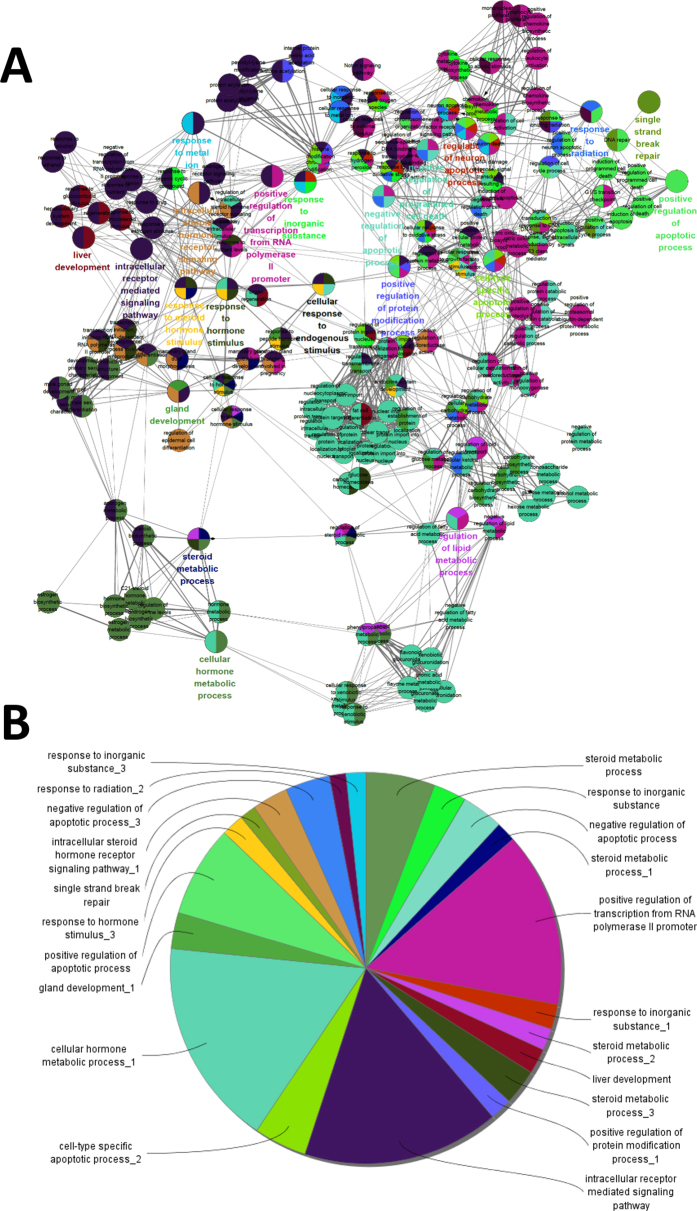
Network groupings based on functionally enriched BP terms. (**A**) A functionally grouped network of enriched categories was generated for AA-related targets using GO terms as nodes and linked using ClueGO analysis. Only the most significant terms in the group are labelled. Functionally related groups partially overlap. (**B**) Functional groups and their corresponding colours.

To annotate the biological functions of AA-related targets, we manipulated the functional enrichment analyses using Gene Ontology (GO) terms and further evaluated the biological processes (BPs) term using the Cytoscape plug-in ClueGO. Overall, 178 GO terms were significantly enriched, as shown in Fig. 5A. These GO terms have been categorised into 21 sub-groups, as shown in Fig. 5B, which primarily involve in liver development, positive regulation of apoptotic process, and cell-type specific apoptotic process.

Many key processes and key factors affected by AA were highlighted by the BP enrichment analyses. We identified the following BPs associated with AA-related targets and their involvement: positive/negative regulation of apoptotic process, negative regulation of programmed cell death, steroid metabolic, liver development, and cellular hormone metabolic. Numerous studies have demonstrated that AA affected these biological processes. Apoptosis is a biological process that responds to toxicity-induced DNA damage. AA exposure induced apoptotic activities in cell culture and in kidney tubular epithelial cells^58^,^59^. Apoptosis is considered as the primary mechanism involved in the development of AAN. AA also induces TEC death *via* apoptosis in acute AAN by targeting the p53 signalling pathway; AA has also been observed to trigger STAT3 dephosphorylation and p53 activation to mediate TEC death *via* the apoptosis mechanism in acute AAN^60^,^61^. While AA can influence steroid and lipid metabolism in the liver^58^ as it inhibits phospholipase A2, which can form fatty acid and lysophospholipid products by hydrolysing phospholipids^62^. AA can significantly inhibit triglyceride accumulation, decrease total cholesterol (TC) and low-density lipoprotein cholesterol (LDL-C) concentrations in the blood, and induce low PPARG (PPAR-γ) expression^57^. These differential biological processes enable us to better understand of the toxicological and pharmacological effects of AA.

#### Schematic construction of an AA target pathway network

**Figure 6:**
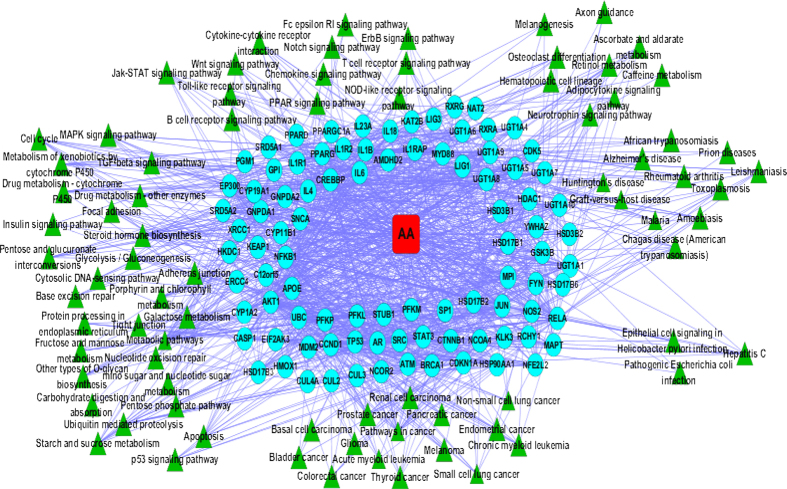
AA target pathway network mode. A red square denotes AA, blue circles denote genes, and green triangles denote KEGG pathways.

To better understand the biological functions of AA-related targets, we carried out pathway-based functional enrichment analyses via WebGestalt. Ninety-one genes were significantly enriched, and 79 significant pathways with cut off P-values <0.05 were systematically enriched. Collectively, an AA target pathway network was constructed in Cytoscape that included 171 nodes and 1299 edges (Fig. 6). Furthermore, the AA target pathway network was divided into approximately 6 parts according to the pathway information.

#### AA causes renal toxicity

Previous studies have demonstrated that AA and its derivatives can affect renal physiological processes by perturbing many molecular pathways, including signal transduction pathways, which lead to the occurrence of tissue toxicity. AA-related targets mostly involved in numerous biological functions that basically accounted for the different stages of renal toxicity. These biochemical functions primarily include AA metabolism, the cell cycle, and the apoptosis pathway as AA induced apoptosis in renal tubular epithelial cells and AAI induced DNA damage and cell cycle arrest in renal tubular epithelial cells through targeting a wild-type p53-independent pathway^63^,^64^,^65^. Here, our investigation of the molecular mechanisms of AA-induced toxicity may explain why apoptosis or limited proliferation and regeneration, the clinical manifestations of AA-associated nephrotoxicity, were induced in tubular epithelial cells.

Many studies have suggested that AA upregulates TGF-β expression, which may account for the development of AAN, and that AA can activate the Smad3 signalling pathway to mediate renal fibrosis by targeting both the TGF-β pathway and the JNK/MAP kinase-dependent pathway^66^,^67^. These studies suggested that the TGF-β/Smad3 signalling pathway may be a novel therapeutic target for chronic AAN.

In addition, as we known that xenobiotic metabolism is associated with the cytochrome P450 metabolic pathway. A line of studies have illustrated that cytochrome P450 (CPY) enzymes mediate the formation of AA-DNA adducts, that the role of this pathway in AAI metabolism *in vivo* has been validated, and that CPY1A1 and CPY1A2 are involved in the detoxification of carcinogenic AA^68^,^69^,^70^. Herein, AA mostly probably affect cytochrome P450 and drug metabolic pathways during the development of renal injury and cancer. Furthermore, AA also exerts substantial influences on some carbohydrate metabolic pathways such as galactose metabolism, fructose and mannose metabolism. Chen *et al.*^5^ have confirmed that AA induces remarkable alterations in carbohydrate metabolism in HK-2 cells. Thus, these carbohydrate metabolic pathways further suggested that AA can induce nephrotoxicity.

#### Anti-inflammatory function of AA

Inflammatory processes are often induced by the actions of pro-inflammatory cytokines, including NFKB1, NFKB3, IL1B, IL18, and IL6, and are resolved by anti-inflammatory cytokines such as IL4 and IL1R2. JAK-STAT signalling can be activated by unregulated cytokines and by cytokine/receptor interactions (JAK-STAT signalling is involved in cyclophosphamide-induced bladder inflammation in female rats). This evidence demonstrates that chronic AAN inflammation can be identified by T cell and macrophage infiltration and by pro-inflammatory chemokine/cytokine upregulation, accompanied by RELA (NFKB3/p65) activation^71^ (NFKB1 and RELA (NFKB3/65) encode the major component of the NF-κB complex). Chen *et al.*^5^ demonstrated that AA can suppress NF-κB activity in human cells, which partly explained the toxicological and anti-inflammatory effects of AA. Toll-like receptors (TLRs) are the primary constituents of the innate immune system that activate inflammatory signalling, including NF-kB activation, which can activate NF-κB *via* a MyD88-dependent signalling pathway^72^,^73^. Moreover, the Wnt signalling pathway participates in many inflammatory responses. Wnt acts *via* the TLR/NF-κB signalling pathway to upregulate pro-inflammatory genes^74^. Wnt also plays a vital role in the AA-induced epithelial to mesenchymal transition (EMT). Moreover, the TLR, NF-κB and Jak/STAT signalling pathways are involved in renal inflammation as well^75^. NF-κB may expand the inflammatory response and plays a core role in renal inflammation.

#### Carcinotoxicity of AA

The molecular mechanism of AA-induced cancer provides a strong association between DNA adducts, p53 mutations and tumour development^2^. Schmeiser *et al.*^76^ revealed that the long-term oral administration of AAI to male Wistar rats has been observed to induce the tumours in multiple organs as they found manifested that AAI induced pancreatic cancer, renal cell carcinoma, lung cancer and other cancers, suggesting that AA-DNA adducts are critical lesions for tumour development. AA-induced A:T-to-T:A transversions are considered as the primary cause of bladder cancer^77^. The ras gene family is primarily involved in cell growth and cell differentiation, and activated ras genes are expressed in colorectal cancer, pancreatic tumours, and thyroid tumours. It was reported that aristolochic acid activates ras genes in rat tumours^78^; whereas AA-mediated induction of colorectal cancers and thyroid tumours may occur by targeting H-ras mutagenic activity.

#### Anti-microorganism activity of AA

AA inhibits the Ca^2+^- and heat shock-induced transformation of *Escherichia coli* with plasmid DNA^79^. Kevekordes *et al.* also confirmed that AA is capable of suppressing *E. coli* growth^80^. Hepatitis C virus (HCV) exposure often leads to severe liver diseases and that p53 is involved in the inhibition of HCV replication in hepatocytes^81^. AA can induce TP53 overexpression that often results in the inhibition of HCV replication. In addition, *Helicobacter pylori* infection induces IL1B expression and NF-κB activation in gastric epithelial cells^82^, it suggested that AA may inhibit *Helicobacter pylori* infection in gastric epithelial cells by blocking NF-κB pathways.

#### Novel biological functions of AA

As a TCM, AA has been broadly utilised for treating many inflammatory diseases such as rheumatoid arthritis. Inflammatory reactions play significant roles in the progressive deterioration observed in Alzheimer’s disease (AD), and NF-κB is the first gene in the inflammation signalling pathway^83^. Recent studies have demonstrated that the dysregulation of the NF-κB pathway induces low HTT expression, which is usually caused by Huntington’s disease (HD) immune dysfunction, and the p65 (RELA/NFKB3)-mediated inflammatory response in astrocytes plays a key role in HD^84^,^85^. Therefore, AA is able to suppress NF-κB activity, which partly clarified the mechanism by which AA holds the capacity to treat AD and HD. Moreover, we also speculated that AA may treat parasitic diseases such as malaria^86^, Chagas disease, leishmaniasis, amoebiasis, toxoplasmosis, and African trypanosomiasis, as well as other diseases such as graft-versus-host disease and prion diseases in terms of its functions with targeting multiple pathways.

Furthermore, we discovered several meaningful pathways related to AA functions that have scarcely been investigated before. These pathways may exert their joint efforts in supporting the system effects of AA in a variety of pathological contexts, such as the neurotrophin signalling pathway, haematopoietic cell lineage, caffeine metabolism, and osteoclast differentiation.

## Conclusion

In the present study, we employed a joint network analysis that comprehensively combines network pharmacology, a protein-protein interaction (PPI) database and a functional annotation analysis to better determine the system effects of AA in terms of tissue toxicity and therapeutic efficiency. The bioinformatics screening of potential protein targets of AA identified a series of candidate protein targets, which were used to construct an AA-specific gene network. These genes are either directly or indirectly associated with AA as determined by literature retrieval and network analyses, furthermore some of new identified genes have been experimentally verified whose expression was significantly modulated by AA treatment with observed dependence of concentration, which basically argued that the developed joint network approach was feasible and robust with the applicable potential to predict the functions of the known and unknown genes in the defined biological events/processes. Moreover, the biological process analysis demonstrated that AA may influence steroid and lipid metabolism, kidney tubular epithelial cells and the inflammatory response. Moreover, we constructed a novel modelling system by integrating multidimensional AA-related targets and pathway information. This system indicated that the multidimensional regulatory effect of AA depended on the combined mechanisms involving nephrotoxicity, anti-inflammation, anti-cancer and anti-microorganism activities, and some other novel regulatory functions. Take altogether, our results suggested that AA exerts multi-target toxicity and broad pharmacological effects during intervening host-pathogen interactions.

## Additional Information

**How to cite this article**: Nie, W. *et al.* Prediction and Characterisation of the System Effects of Aristolochic Acid: A Novel Joint Network Analysis towards Therapeutic and Toxicological Mechanisms. *Sci. Rep.* **5**, 17646; doi: 10.1038/srep17646 (2015).

## Figures and Tables

**Table 1:** Fifteen protein targets associated with AA.

No	Protein Targets	Gene Symbol
1	oestrogen nuclear receptor alpha	ESR1
2	glucocorticoid receptor	NR3C1
3	cellular tumour antigen p53	TP53
4	sentrin-specific protease 8	SENP8
5	nuclear factor erythroid 2-related factor 2 isoform 2	NFE2L2
6	peroxisome proliferator-activated receptor gamma	PPARG
7	peroxisome proliferator-activated receptor delta	PPARD
8	putative hexokinase HKDC1	HKDC1
9	AR protein cytochrome P450, family 19, subfamily A, polypeptide 1, isoform CRA a	AR
10	cytochrome P450, family 19, subfamily A, polypeptide 1, isoform	CYP19A1
11	microtubule-associated protein tau	MAPT
12	TDP1 protein	TDP1
13	cytochrome P450 1A2	CYP1A2
14	nuclear factor erythroid 2-related factor 2 isoform 1	NFE2L2
15	interleukin-1 beta proprotein	IL1B

**Table 2:** Primer sequence for the targeted genes expressed in HK-2 cells.

Gene Symbol	Primer F	Primer R
AR	5′-GCCTGGCTTCCGCAACTTACAC-3′	5′-GCGAAGTAGAGCATCCTGGAGT-3′
C11ORF17	5′-CCCCAACCCTTAGTGCTTCCTTC-3′	5′-GCTTCGACTCGCCTCTGTGATA-3′
CDK5	5′-CAATGGTGACCTCGATCCTGAG-3′	5′-CCTGTTTATTAGCGGGTTCTGG-3′
CDKN1A	5′-TCACCGAGACACCACTGGAGGG-3′	5′-CCTGAGCGAGGCACAAGGGTAC-3′
CYP19A1	5′-TTTTGGAAATGCTGAACCCGATAC-3′	5′-GTAGTTGCAGGCACTGCCGATC-3′
ESR	5′-CATGAAGTGCAAGAACGTGGTG-3′	5′-AAGGAATGCGATGAAGTAGAGCC-3′
ESRRA	5′-GTGGGCGGCAGAAGTACAAG-3′	5′-TCGGTCAAAGAGGTCACAGAGGGT-3′
ILI8	5′-TAAAGATAGCCAGCCTAGAGGTAT-3′	5′-TGTTATCAGGAGGATTCATTTC-3′
IL1R1	5′-ATACTTGGGCAAGCAATATCCT-3′	5′-TGTCTCATTAGCTGGGCTCACA-3′
IL1RAP	5′-CTCTGACTGTAAAGGTAGTAGGCTCT-3′	5′-TTCCATCAATGGTCCACCAAAC
IL23A	5′-TCTGCTCCCTGATAGCCCTGTG-3′	5′-CTTGGAATCTGCTGAGTCTCC-3′
IL4	5′-TTCTCTGCTCCCTGATAGCC-3′	5′-CTTGGAATCTGCTGAGTCT-3′
JUN	5′-CGGTCTACGCAAACCTCAGCAACT-3′	5′-TGATCCGCTCCTGGGACTCCAT-3′
GAPDH	5′-TCCCTGAGCTGAACGGGAAG-3′	5′-GGAGGAGTGGGTGTCGCTGT-3′
